# Supplementary material for: Comparative LCA of diesel and grid electricity for agricultural irrigation in Iran: why diesel outperforms in fossil-reliant grids
Source: PLoS One. 2025 Jul 23;20(7):e0324371. doi: 10.1371/journal.pone.0324371 (PMC12286319; doi:10.1371/journal.pone.0324371)
Supplement: Attachment — Submitted filename: Response to Reviewers.docx [file pone.0324371.s003.docx]

**Response to Reviewers**

**Dear Editors and Reviewers,**

Thank you very much for your valuable feedback and constructive comments on our manuscript titled "**Comparative LCA of diesel and grid electricity for agricultural irrigation in Iran: why diesel outperforms in fossil-reliant grids**" We viewers’ comments and outline the changes made to the manuscript.

**Reviewer #1:**

**Comment 1:** What are the key environmental indicators evaluated in the study using Life Cycle Assessment (LCA) methodology?

Response:

Thank you for your valuable comment. In response to your question regarding the key environmental indicators evaluated in the study, we have added a detailed explanation in the Materials and Methods section of the manuscript. Specifically, we have included a new paragraph (highlighted in yellow) that lists and describes the environmental impact categories assessed using the CML methodology. These categories include:

- Abiotic Depletion (ADP)
- Abiotic Depletion (Fossil Fuels) (ADF)
- Global Warming Potential (GWP)
- Ozone Layer Depletion Potential (ODP)
- Human Toxicity (HT)
- Freshwater Aquatic Ecotoxicity (FWAE)
- Marine Aquatic Ecotoxicity (MAE)
- Terrestrial Ecotoxicity (TE)
- Photochemical Oxidation (PO)
- Acidification Potential (AP)
- Eutrophication Potential (EP)

We believe this addition provides a clearer and more comprehensive explanation of the environmental indicators evaluated in our study, addressing your concern directly. Please find the revised text in the updated manuscript.

**Comment 2:** How does the Environmental Composite Index (ECI) of diesel-powered well water extraction systems compare to grid-connected electricity systems?

Response:greatly appreciate the time and effort you have dedicated to reviewing our work. We have carefully considered all the comments and suggestions, and have made the necessary revisions to improve the quality of the manuscript. Below, we provide a point-by-point response to the re

Dear Reviewer, Thank you for your valuable comment regarding the comparison of the Environmental Composite Index (ECI) between diesel-powered and grid-connected electricity systems. We would like to highlight that this comparison and its underlying reasons are already discussed in detail in the Results and Discussion section of the manuscript. Specifically, please refer to the following parts:

Section 3.3 (Comparative Analysis of Environmental Impacts: Diesel vs. Electricity-Powered Systems): This section provides a comprehensive comparison of the ECI values for both systems, highlighting that the diesel-powered system has a lower ECI (3.68 × 10^-4^ nPt) compared to the grid-connected system (5.77 × 10^-4^ nPt), representing a 56.79% lower environmental burden for diesel systems.

Section 3.4 (Effects of Local and Technical Parameters on Environmental Impacts of Irrigation Pumping Systems): This section explains the reasons for this difference, including the heavy reliance on fossil fuels in Iran's electricity grid, the absence of transmission losses in diesel systems, and the inefficiencies of Iran's power generation infrastructure.

We believe that these sections address your question thoroughly. However, to make this information more accessible, we have highlighted the relevant paragraphs in yellow in the revised manuscript.

Thank you again for your insightful comment, which has helped us ensure that the key findings of our study are clearly communicated.

**Comment 3:** What percentage higher environmental burden is associated with grid electricity systems compared to diesel-powered systems?

Response:

The grid-connected electricity system exhibits a 56.79% higher environmental burden compared to the diesel-powered system, as measured by the Environmental Composite Index (ECI). Specifically, the ECI for the grid-connected system is 5.77 × 10^-4^ nPt, while the ECI for the diesel-powered system is 3.68 × 10^-4^ nPt. This difference highlights the significant environmental impact associated with grid electricity, primarily due to Iran's heavy reliance on fossil fuels for electricity generation and the inefficiencies in power transmission and distribution.

**Comment 4:** What are the primary drivers of environmental impacts in grid-connected electricity systems according to the study?

Response:

Thank you for your valuable comment regarding the primary drivers of environmental impacts in grid-connected electricity systems. In response to your question, we have added a detailed explanation in the Results and Discussion section of the manuscript. Specifically, we have included a new paragraph (highlighted in yellow) that identifies and discusses the key factors contributing to the environmental impacts of grid-connected systems. These factors include:

- The heavy reliance on fossil fuels for electricity generation in Iran, which results in significant emissions of greenhouse gases (GHGs) and other pollutants.
- Transmission and distribution losses, which exacerbate the environmental burden.
- The composition of the electricity grid, dominated by fossil fuels, and its influence on impact categories such as Global Warming Potential (GWP), Abiotic Depletion (Fossil Fuels) (ADF), and Marine Aquatic Ecotoxicity (MAE).
- The inefficiencies of Iran's fossil fuel-based power plants, which further contribute to the environmental burden.

We believe this addition provides a clearer and more comprehensive explanation of the primary drivers of environmental impacts in grid-connected systems, addressing your concern directly. Please find the revised text in the updated manuscript.

Thank you again for your insightful comment, which has helped improve the clarity of our manuscript.

**Comment 5:** Which environmental impact categories do grid electricity systems exhibit higher impacts in compared to diesel systems?

In which impact categories do diesel systems demonstrate greater impacts compared to grid electricity systems?

Response:

Thank you for your valuable comment regarding the comparison of environmental impact categories between grid-connected electricity systems and diesel-powered systems. We would like to highlight that this comparison is already discussed in detail in the Results and Discussion section of the manuscript. Specifically, please refer to the following parts:

Section 3.3 (Comparative Analysis of Environmental Impacts: Diesel vs. Electricity-Powered Systems): This section provides a comprehensive comparison of the environmental impact categories for both systems, highlighting the categories where grid-connected systems exhibit higher impacts (e.g., Abiotic Depletion, Global Warming Potential, and Marine Aquatic Ecotoxicity) and those where diesel systems demonstrate greater impacts (e.g., Ozone Layer Depletion, Human Toxicity, and Acidification Potential).

We believe that these sections address your question thoroughly. However, to make this information more accessible, we have highlighted the relevant paragraphs in yellow in the revised manuscript.

Thank you again for your insightful comment, which has helped us ensure that the key findings of our study are clearly communicated.

**Comment 6:** How do local and technical factors, such as energy source composition and power plant efficiency, influence environmental outcomes in well water extraction systems?

Response:

Thank you for your valuable comment regarding the influence of local and technical factors on the environmental outcomes of well water extraction systems.

Section 3.4 (Effects of Local and Technical Parameters on Environmental Impacts of Irrigation Pumping Systems): This section provides a comprehensive analysis of how factors such as energy source composition, power plant efficiency, transmission and distribution losses, and pump efficiency influence the environmental performance of both diesel-powered and grid-connected systems.

we have highlighted the relevant paragraphs in yellow in the revised manuscript.

Thank you again for your insightful comment

**Comment 7:** Revise the text by native English editor

Response:

Thank you for your valuable comment regarding the need for language editing. We acknowledge the importance of ensuring that the manuscript is written in clear and fluent English. In response to your suggestion, we have engaged a professional native English editor to revise the entire manuscript. The editor has carefully reviewed the text to improve grammar, sentence structure, and overall readability while ensuring that the scientific content remains accurate and unchanged.

We believe that these revisions have significantly enhanced the quality of the manuscript. The edited version is now ready for your review, and we hope that it meets the journal's standards for language and clarity.

**Comment 8:** What strategies for environmental mitigation are suggested by the study to reduce the impacts of well water extraction systems?

Response:

Thank you for your valuable comment regarding the strategies for environmental mitigation to reduce the impacts of well water extraction systems. In response to your question, we have added a detailed explanation in the Results and Discussion section of the manuscript. Specifically, we have included a new paragraph (highlighted in yellow) that outlines the key mitigation strategies proposed by the study. These strategies include:

Integration of renewable energy sources (e.g., solar and wind) to power water extraction systems.

- Modernization of power generation infrastructure to improve efficiency.
- Enhancement of pump efficiency through technological upgrades.
- Policy interventions, such as subsidies for renewable energy systems and penalties for inefficient pumps.
- Improved water management practices, such as drip irrigation and precision agriculture.
- Investment in research and development to explore innovative solutions for sustainable water extraction and energy use.

We believe this addition provides a clearer and more comprehensive explanation of the proposed mitigation strategies, addressing your concern directly. Please find the revised text highlighted in yellow in the updated manuscript.

**Comment 9:** The below references can be used in the introduction section to improve it:

- Guo, H., Li, Y., Zhao, B., Guo, Y., Xie, Z., Morina, A.,... Lu, X. (2024). Transient lubrication of floating bush coupled with dynamics and kinematics of cam-roller in fuel supply mechanism of diesel engine. Physics of Fluids, 36(12), 123103. doi: <https://doi.org/10.1063/5.0232226>
- Chen, L., Cui, B., Zhang, C., Hu, X., Wang, Y., Li, G.,... Liu, L. (2024). Impacts of Fuel Stage Ratio on the Morphological and Nanostructural Characteristics of Soot Emissions from a Twin Annular Premixing Swirler Combustor. Environmental Science & Technology, 58(24), 10558-10566. doi: <https://doi.org/10.1021/acs.est.4c03478>
- Chen, L., Cao, Y., Hu, X., Zhang, B., Chen, X., Cui, B.,... Xu, Z. (2025). Raman spectral optimization for soot particles: A comparative analysis of fitting models and machine learning enhanced characterization in combustion systems. Building and Environment, 271, 112600. doi: <https://doi.org/10.1016/j.buildenv.2025.112600>
- Qiu, Z., Chen, R., Gan, X., & Wu, C. (2024). Torsional damper design for diesel engine: theory and application. Physica Scripta, 99(12), 125214. doi: 10.1088/1402-4896/ad8af8
- Zhang, Z., Bu, Y., Wu, H., Wu, L., & Cui, L. (2023). Parametric study of the effects of clump weights on the performance of a novel wind-wave hybrid system. Renewable Energy, 219(Part 1), 119464. doi: <https://doi.org/10.1016/j.renene.2023.119464>

Response:

Thank you for your valuable suggestion regarding the inclusion of additional references to improve the introduction section. We appreciate your recommendations and have carefully reviewed the suggested references. In response to your comment, we have incorporated these references in the manuscript. We believe that these additions have enhanced the quality and depth of the manuscript.

**Reviewer #2:**

**Comment 1: Introduction & Literature Review**

The introduction should better contextualize the study within recent global trends in renewable energy adoption for irrigation (e.g., solar/wind). Expand the literature review to include studies from regions with similar energy grids (e.g., Middle East) to strengthen the rationale for comparing diesel and grid electricity in Iran. I recommend to apply the below reference in this section:

1. Yao, Y., Sheng, H., Luo, Y., He, M., Li, X., Zhang, H., ... & An, L. (2014). Optimization of anaerobic co-digestion of Solidago canadensis L. biomass and cattle slurry. Energy, 78, 122-127.
2. Yao, Y., Bergeron, A. D., & Davaritouchaee, M. (2018). Methane recovery from anaerobic digestion of urea-pretreated wheat straw. Renewable energy, 115, 139-148.
3. Deng, Y., Qiu, L., Shao, Y., & Yao, Y. (2019). Process modeling and optimization of anaerobic Co-digestion of peanut hulls and swine manure using response surface methodology. Energy & Fuels, 33(11), 11021-11033.
4. Qiu, L., Deng, Y. F., Wang, F., Davaritouchaee, M., & Yao, Y. Q. (2019). A review on biochar-mediated anaerobic digestion with enhanced methane recovery. Renewable and Sustainable Energy Reviews, 115, 109373.
5. Wu, H., Li, A., Zhang, H., Li, S., Yang, C., Lv, H., & Yao, Y. (2024). Microbial mechanisms for higher hydrogen production in anaerobic digestion at constant temperature versus gradient heating. Microbiome, 12(1), 170.
6. Zhang, Y., Xu, L., Wang, J., Pan, H., Dou, M., Teng, Y., ... & Wang, M. (2024). Bagasse-based porous flower-like MoS2/carbon composites for efficient microwave absorption. Carbon Letters, 1-16.
7. Yang, Z. Q., & Zhu, H. L. (2023). Study on the effect of carbon nanotubes on the microstructure and anti-carbonation properties of cement-based materials. Journal of Functional Materials, 54(08), 8217-8227.

Response:

Thank you for your valuable comment regarding the need to better contextualize the study within recent global trends in renewable energy adoption for irrigation and to expand the literature review to include studies from regions with similar energy grids. In response to your suggestion, we have revised the Introduction section. We appreciate your recommendations and have carefully reviewed the suggested references. In response to your comment, we have incorporated these references in the manuscript.

**Comment 2: Methodology: LCA Approach & Data Representativeness**

Clarify why the CML methodology and SimaPro software were chosen over other LCA frameworks (e.g., ReCiPe). Justify the sample size (100 wells) and geographic distribution (Tehran, Hamadan, etc.) to ensure representativeness. Address potential biases in data collection (e.g., self-reported diesel use vs. metered electricity).

Response:

Thank you for your valuable comments regarding the methodology of our study. Below, we address each of your concerns in detail:

1- Justification for Choosing the CML Methodology and SimaPro Software:

The CML methodology was selected for this study due to its objectivity and international recognition in environmental impact assessment. Unlike other frameworks such as ReCiPe, which incorporate subjective weighting factors based on societal preferences, the CML methodology relies on scientific and objective criteria for impact assessment. This makes it particularly suitable for comparative studies like ours, where the goal is to provide an unbiased evaluation of environmental impacts (Dekamin et al., 2018; Rasooli et al., 2023). Additionally, the CML methodology is well-documented and widely used in LCA studies, ensuring consistency and comparability with other research.

The SimaPro software was chosen because it is one of the most comprehensive and widely used tools for LCA studies. It supports the CML methodology and provides a robust platform for modeling complex systems, such as diesel-powered and grid-connected irrigation systems. SimaPro's extensive database and flexibility in handling large datasets make it ideal for this study.

2- Justification for Sample Size and Geographic Distribution:

The sample size of 100 wells was determined based on the need to achieve a representative and statistically robust dataset while considering practical constraints such as data availability and accessibility. This sample size is sufficient to capture the variability in energy consumption and environmental impacts across different regions and well types.

The geographic distribution of wells across Tehran, Hamadan, Kurdistan, and Fars was chosen to ensure regional diversity and representativeness. These provinces were selected because they cover a wide range of climatic conditions, agricultural practices, and energy infrastructure, which are critical factors influencing the environmental performance of irrigation systems. By including wells from these regions, we aimed to provide a comprehensive analysis that reflects the diversity of Iran's agricultural landscape.

3- Addressing Potential Biases in Data Collection:

We acknowledge the potential for biases in data collection, particularly regarding self-reported diesel use versus metered electricity consumption. To mitigate these biases, we implemented the following measures:

- Diesel Consumption Data: Diesel use was verified through fuel purchase records and interviews with farmers, ensuring accuracy and reliability.
- Electricity Consumption Data: Electricity use was measured using meter readings installed at the wells, providing precise and objective data.

**Comment 3: System Boundaries & Energy Mix Assumptions**

Explicitly define system boundaries for both scenarios (e.g., inclusion/exclusion of transmission losses, pump maintenance). Discuss how Iran’s electricity grid composition (94% fossil fuels, per Table 8) was modeled, and sensitivity analysis for varying energy mixes (e.g., renewables) should be included.

Response:

Thank you for your valuable comments regarding the system boundaries, modeling of Iran's electricity grid composition, and the need for sensitivity analysis. Below, we address each of your concerns in detail:

**1. Explicit Definition of System Boundaries:**

The system boundaries for both scenarios were defined using a cradle-to-use approach, encompassing all stages from energy production to water extraction. Below, we clarify the inclusion and exclusion of key processes for each scenario:

*Diesel-Powered Systems (Scenario I):*

- **Included Processes:**
- Diesel fuel production (extraction, refining, and transportation).
- Diesel fuel distribution and storage.
- On-farm diesel combustion for water extraction.
- Emissions from diesel combustion (e.g., CO_2_, NO_x_, …).
- Manufacturing and disposal of diesel engines and pumps (included due to their potential environmental impacts, despite their long lifespan).
- **Excluded Processes:**
- Maintenance activities (e.g., pump repairs).

*Grid-Connected Electricity Systems (Scenario II):*

- **Included Processes:**
- Electricity generation in power plants.
- Transmission and distribution losses.
- Electricity consumption for water extraction.
- Infrastructure for electricity distribution (e.g., power poles, transformers, cables).
- Manufacturing of electrical equipment (e.g., pumps, transformers).
- **Excluded Processes:**
- Maintenance activities (e.g., pump repairs, grid maintenance).

**2- Modeling Iran’s Electricity Grid Composition and Environmental Impacts:**

Iran's electricity grid composition, which is 94% reliant on fossil fuels, was modeled using official statistics from the International Energy Agency (IEA) for the year 2025. This data ensures that our analysis accurately reflects the current and projected energy mix in Iran. The environmental impacts of electricity generation were calculated based on the emissions and resource consumption associated with each energy source.

**3- Sensitivity Analysis for Varying Energy Mixes:**

A sensitivity analysis was conducted to evaluate the impact of varying energy mixes on environmental outcomes.

All the requested changes have been incorporated into the revised version of the manuscript (highlighted in green).

**Comment 4: Results Presentation & Figure Clarity**

Revise Figures 5–11 for clarity (e.g., label axes, simplify color schemes). Ensure consistency in units (e.g., nPt vs. kg CO₂-eq) and provide error bars/uncertainty ranges for impact categories. Highlight key drivers (e.g., marine ecotoxicity in diesel systems) in figure captions.

Response:

Thank you for your detailed feedback regarding Figures 5–11. To enhance the clarity and interpretability of the results, Figures 5–11 have been revised as follows:

- All axes are now clearly labeled with appropriate units, and color schemes have been simplified for better readability.
- Units have been standardized across all figures, with impacts expressed in nPt for normalized and weighted results and specific impact categories (e.g., GWP) expressed in kg CO₂-eq.
- Error bars or uncertainty ranges have been added to reflect the variability in the data, calculated based on the standard deviation of the data points.
- Figure captions have been revised to highlight key drivers of environmental impacts, such as the significant contribution of marine aquatic ecotoxicity in diesel-powered systems and the dominant role of electricity generation in grid-connected systems.

**Comment 5: Interpretation of Key Findings**

Strengthen the discussion of why diesel outperforms grid electricity in Iran (e.g., direct energy conversion vs. grid inefficiencies). Compare results with studies in similar contexts (e.g., Pradeleix et al. 2015 for Tunisia) and explain discrepancies (e.g., El-Gafy & El-Bably 2016 for Egypt).

Response:

Thank you for your insightful comment regarding the comparison of diesel and grid electricity performance in Iran. In response to your suggestions, we have strengthened the discussion in the Results and Discussion section as follows:

**1. Why Diesel Outperforms Grid Electricity in Iran:**

The superior performance of diesel-powered systems in Iran can be attributed to several factors:

Direct Energy Conversion: Diesel systems convert fuel directly into mechanical energy on-site, avoiding the significant energy losses associated with electricity transmission and distribution in grid-connected systems.

Grid Inefficiencies: Iran's electricity grid is heavily reliant on fossil fuels (94%), and the inefficiencies in power generation, transmission, and distribution exacerbate the environmental burden of grid-connected systems.

On-Farm vs. Off-Farm Emissions: While diesel systems generate on-farm emissions, grid-connected systems produce off-farm emissions during electricity generation, which are often more significant due to the centralized nature of power plants.

These factors collectively explain why diesel systems exhibit a lower Environmental Composite Index (ECI) compared to grid-connected systems in the Iranian context.

**2. Comparison with Studies in Similar Contexts:**

Our findings align with some studies but differ from others, depending on the local energy mix and technical conditions:

Pradeleix et al. (2015) for Tunisia: Similar to our study, Pradeleix et al. found that diesel systems can outperform grid-connected systems when the electricity grid relies heavily on fossil fuels. However, they also noted that diesel systems become less favorable when grid electricity is generated from cleaner sources (e.g., natural gas).

El-Gafy & El-Bably (2016) for Egypt: In contrast to our findings, El-Gafy & El-Bably reported that grid-connected systems outperformed diesel systems in Egypt. This discrepancy can be attributed to differences in the energy mix (Egypt has a higher share of natural gas in its electricity grid) and pump efficiencies.

**3. Explanation of Discrepancies:**

The differences between our results and those of other studies can be explained by:

Energy Mix: The environmental performance of grid-connected systems is highly dependent on the energy mix. In regions with a higher share of renewables or natural gas, grid-connected systems tend to perform better.

Technical Factors: Variations in pump efficiency, transmission losses, and power plant efficiency can significantly influence the results.

Local Conditions: Factors such as climate, water availability, and agricultural practices also play a role in shaping the environmental outcomes of irrigation systems.

**Comment 6: Limitations & Future Research**

Expand the limitations section to address:

• Lack of renewable energy systems in the analysis.

• Assumptions about pump efficiency (e.g., outdated diesel engines).

• Geographic specificity of Iran’s grid. Suggest future studies on hybrid systems (e.g., solar-diesel).

Response:

We thank the reviewer for their valuable suggestions. In response, we have expanded the Conclusion section to include a detailed discussion of limitations and future research directions. Specifically, we have:

1. Acknowledged the exclusion of renewable energy systems and emphasized the need for future studies to incorporate solar, wind, and hybrid systems.
2. Discussed the assumptions about pump efficiency and highlighted the potential benefits of upgrading to more efficient technologies.
3. Addressed the geographic specificity of Iran’s grid and suggested future research in regions with diverse energy mixes.
4. Proposed the exploration of hybrid systems (e.g., solar-diesel) as a promising direction for future research.

These changes have been incorporated into the revised manuscript (see Section 4: Conclusion).

**Comment 7: Conclusion Overgeneralization**

Revise the conclusion to emphasize that the diesel advantage is context-specific to Iran’s fossil-reliant grid. Avoid implying universal applicability; instead, frame recommendations for regions with similar energy profiles.

Response:

We sincerely thank the reviewer for their insightful comment regarding the overgeneralization in the conclusion. We fully agree that the environmental advantage of diesel-powered systems observed in this study is specific to Iran's fossil fuel-dependent electricity grid and should not be implied as universally applicable.

To address this concern, we have made the following revisions to the conclusion:

Context-Specific Emphasis: We have explicitly stated that the observed advantage of diesel-powered systems is tied to Iran's current energy context, particularly its reliance on fossil fuels for electricity generation.

Avoiding Universal Applicability: We have removed any language that could imply universal applicability and clarified that the findings are specific to regions with similar energy profiles.

Tailored Recommendations: We have framed our recommendations to focus on regions with energy grids heavily reliant on fossil fuels, ensuring that the conclusions remain relevant and context-appropriate.

These changes ensure that the conclusion accurately reflects the context-specific nature of the findings while providing actionable insights for regions with comparable energy profiles.

**Comment 8: Renewable Energy Integration**

Discuss policy implications for transitioning to renewables (e.g., solar pumps) given Iran’s high solar potential. Reference studies like Guno (2024) and Terang & Baruah (2023) to support this.

Response:

We sincerely thank the reviewer for their insightful suggestion regarding the integration of renewable energy, particularly solar-powered irrigation systems, in the context of Iran’s high solar potential. This is an important aspect that strengthens the discussion on sustainable agricultural practices, and we have revised the manuscript accordingly.

To address this comment, we have added a detailed discussion on the policy implications of transitioning to renewable energy systems, specifically solar-powered water pumps, while referencing the findings of Guno (2024) and Terang & Baruah (2023). The new text has been incorporated at the end of the Discussion section and is highlighted in green for ease of reference.

**Comment 9: Language & Technical Precision**

Proofread for grammatical errors (e.g., "electricity system’s" vs. "electricity systems"). Use consistent terminology (e.g., "abiotic depletion" vs. "resource depletion") and define acronyms (e.g., ECI, FU) at first use.

Response:

We sincerely thank the reviewer for their valuable feedback regarding language and technical precision. All the suggested corrections regarding grammar, terminology consistency, and acronym definitions have been carefully implemented.

**Comment 10: Title & Keyword Specificity**

Revise the title to reflect Iran’s context and key findings: “Comparative LCA of Diesel and Grid Electricity for Irrigation in Iran: Why Diesel Outperforms in Fossil-Reliant Grids.”

Add keywords like “Iran,” “groundwater pumping,” and “renewable energy transition.

Response:

Thank you for your valuable feedback; the suggested changes regarding the title and keywords have been successfully incorporated into the final manuscript.
