# Supplementary material for: Comparative LCA of diesel and grid electricity for agricultural irrigation in Iran: why diesel outperforms in fossil-reliant grids
Source: PLoS One. 2025 Jul 23;20(7):e0324371. doi: 10.1371/journal.pone.0324371 (PMC12286319; doi:10.1371/journal.pone.0324371)
Supplement: Attachment — Submitted filename: Response_to_Reviewers_auresp_2.docx [file pone.0324371.s004.docx]

**Response to Reviewers**

**Dear Editors and Reviewers,**

We thank the Academic Editor and both reviewers for their valuable comments and positive evaluation of our revised manuscript. We are grateful for their time and effort, and we have addressed all the comments carefully as follows:

**Reviewer #1:**

*Comment:*

All my comments were addressed and the paper can be accepted in the present form. Congratulations.

*Response:*

We appreciate your positive feedback and are grateful for your acceptance of our revisions.

**Reviewer #2:**

*Comment 1:*

The introduction section needs minor improvement. The following references are suggested to strengthen the innovation aspects of the paper:

- Yu, Z., Zhao, J., Markov, V., & Han, D. (2024). Effects of hydrogen addition on ignition characteristics and engine performance of ammonia-hydrogen blended fuel: A kinetic analysis. International Journal of Hydrogen Energy, 87, 722-735.
- Yao, Y., & Chen, S. (2016). A novel and simple approach to the good process performance of methane recovery from lignocellulosic biomass alone. Biotechnology for biofuels, 9, 1-9.
- Yao, Y., Luo, Y., Yang, Y., Sheng, H., Li, X., Li, T., ... & An, L. (2014). Water free anaerobic co-digestion of vegetable processing waste with cattle slurry for methane production at high total solid content. Energy, 74, 309-313.
- Yang, Z. Q., Hou, K. P., & Guo, T. T. (2011). Study on the effects of different water-cement ratios on the flow pattern properties of cement grouts. Applied Mechanics and Materials, 71, 1264-1267.
- Gao, J., Wu, Y., & Shen, T. (2017). On-line statistical combustion phase optimization and control of SI gasoline engines. Applied Thermal Engineering, 112, 1396-1407.

*Response:*

Thank you for your valuable suggestion. In the revised version of the paper, we have carefully reviewed and incorporated the recommended literature to strengthen the introduction section. These additions have helped us better position our work within the existing research landscape and highlight the novel contributions of our study.

We believe the revised manuscript has significantly improved in clarity and scientific rigor, thanks to your valuable suggestions.

*Comment 2:*

In the text, the citation “Zhang et al., 2025” should be corrected to “Zhang et al., 2024.”

Response:

Thank you for spotting this mistake. We have corrected the citation in the main text to “Zhang et al., 2024.”

*Comment 3:*

Use more results in the abstract section and improve it so that readers can get the needed information.

*Response:*

We have revised the abstract by including more quantitative results and key findings from our analysis to provide a clearer and more informative summary for readers.
